# The Addition of Systemic Terbinafine to Antifungal Combination Therapy in the Treatment of Disseminated Drug-Resistant Mold Infections in a National Cancer Institute Comprehensive Cancer Center: A Six-Year Study

**DOI:** 10.7759/cureus.55343

**Published:** 2024-03-01

**Authors:** David B Thomas, Eloho Ajayi, Anna Sikora, Lisa Cozzini, Rod Quilitz, Yanina Pasikhova, Olga Klinkova, Aliyah Baluch

**Affiliations:** 1 Transplant and Oncology Infectious Diseases, Houston Lee Moffitt Cancer Center & Research Institute, Tampa, USA; 2 Infectious Diseases, Houston Lee Moffitt Cancer Center & Research Institute, Tampa, USA; 3 Infectious Diseases and International Medicine, University of South Florida, Morsani College of Medicine, Tampa, USA; 4 Clinical Pharmacy, Houston Lee Moffitt Cancer Center & Research Institute, Tampa, USA; 5 Clinical Pharmacy, Moffitt Cancer Center, Tampa, USA; 6 Infectious Diseases, Moffitt Cancer Center, Tampa, USA

**Keywords:** invasive fungal infections, combination, hematology-oncology, cancer center, terbinafine

## Abstract

Introduction

Combination antifungal regimens are frequently employed in the treatment of invasive fungal infections in patients who are immunocompromised, particularly for cancer and transplant patients. Terbinafine is a potential agent of interest for combination regimens.

Methods

We reviewed data over a six-year period examining patient outcomes in terms of both mortality and distribution of pathogens. The total number of patients in our study was 64. The use of terbinafine versus no terbinafine in combination therapy was assessed. Of the 64 patients analyzed, only 14 received terbinafine. Mortality was calculated for both groups, and demographics were analyzed by descriptive statistics.

Results

There was no statistical difference in mortality outcomes in either group. The addition of terbinafine was well tolerated and did not appear to result in any undue toxicity concerns.

Discussion

We wish to draw greater attention to this potential agent within our armamentarium for invasive fungal infections. To our knowledge, the total number of patients in our study, while small, represents the largest reported cohort in the literature to date. Sensitivities are crucial to be obtained for fungal pathogens as this likely undermined the utility of terbinafine in our study with larger than expected numbers of multidrug-resistant *Fusarium*. With limited patient numbers, a multicenter trial would be beneficial to further examine terbinafine in combination regimens.

## Introduction

It is estimated that there are approximately 1.5-5 million fungal species globally [1]. A large portion of fungi can be found in the environment, while others are a component of the normal flora of humans and animals [2]. Severely immunocompromised patients, such as those being treated for hematological cancers, and recipients of hematopoietic cell transplantation (HCT) or solid organ transplantation (SOT) are at high risk for acquiring such infections [3,4]. It has been predicted that only several hundred fungi can be pathogenic for humans. Medically relevant mold genera such as *Aspergillus*, *Fusarium*, *Mucor*, *Rhizopus*, *Scedosporium*, and *Curvularia* have been identified as causative agents in the pathogenesis of invasive mold infection (IMI) [5].

Invasive aspergillosis (IA) is currently the most common cause of IMI in immunocompromised patients [6]. Voriconazole has been recommended as primary therapy for IA with lipid formulations of amphotericin B (AmB) being an effective alternative agent [7]. In addition to voriconazole, both posaconazole and isavuconazole are non-inferior in head-to-head FDA clinical trials. With regard to the management of IMI caused by the *Mucorales*, liposomal AmB is the first-line pharmacologic agent used during induction therapy [8]. Isavuconazole and posaconazole are alternative agents for the treatment of mucormycosis, especially in patients with intolerance or refractory infections. There is data to suggest that surgical intervention in SOT recipients with mucormycosis was independently associated with improved survival as well [9].

*Fusarium *spp. causes about 13% of non-aspergillus mold infections in SOT recipients. Several *Fusarium *species are resistant to AmB in vitro, thus voriconazole is generally considered as first-line therapy. However, laboratory data suggests a wide range of susceptibilities to voriconazole among various *Fusarium *species [8]. *Scedosporium *spp. is an example of an emerging medically relevant mold that can cause serious life-threatening disseminated infections in profoundly immunocompromised patients. It is worth noting that AmB has no activity against *Scedosporium apiospermum*, while in vitro data suggests that voriconazole is the most potent agent against this organism [8].

At present, there are no strong evidence-based guidelines regarding the use of combination therapy in the treatment of invasive fungal infections in cancer or hematologic transplant patients. The Infectious Diseases Society of America (IDSA) treatment guidelines for IA, for example, cited that the first-line therapy recommended is single-agent voriconazole [7]. They briefly mention that combination antifungal therapy with voriconazole and an echinocandin may be considered in select patients as a salvage citing a very weak level of evidence. Further, they mention that when patients are treated with combination therapy, the impact of the echinocandin agent is difficult to specifically define. This is largely echoed in the American Transplant Society's guidelines for IA as well [8]. In all instances, combination therapy presumes an echinocandin as a second agent [7,8]. It is understood there is a lack of correlation between clinical outcomes and in-vitro activity with respect to antifungal drugs; as such, susceptibility testing is not often undertaken in many cases [10]. Such an observation is noted in this study as well.

This retrospective study was undertaken to describe the clinical outcomes observed when oral terbinafine is added in combination with other systemic antifungal agents in the treatment of invasive mold infections at a tertiary cancer center. Our study predates the development and clinical use of experimental agents such as orolofilm and fosmanogepix. Terbinafine was chosen as it is an FDA-approved antifungal agent readily available commercially in our country. It has anecdotally demonstrated potential anti-mold synergy with the triazoles and/or amphotericin B in some reports [11,12]. Based on this, we sought to determine if the addition of terbinafine to a combination regimen has yielded improvements in clinical outcomes within our center.

## Materials and methods

The study was a retrospective review of electronic medical records. The study population was composed of patients treated for culture-confirmed infection with mold isolates (n = 64) at the Moffitt Cancer Center from 1/1/2015 to 6/1/2021. Patients were grouped into two treatment groups: Group A patients were treated with a terbinafine-containing regimen, and Group B patients received an open-label (physician's choice) antifungal regimen that did not include terbinafine for the purposes of this study. We compared only the addition of terbinafine in these regimens to its lack thereof. Regimens utilized in combination therapy at our center typically are triazole, most commonly voriconazole, along with the addition of either an echinocandin (micafungin on our institutional formulary) or amphotericin B. Data were obtained through searches in our institution’s antimicrobial stewardship database and the microbiology laboratory database to determine eligible subjects.

Data collected included baseline characteristics including age, gender, race, medical record number, absolute neutrophil count (at time of diagnosis with IMI), duration of neutropenia (if neutropenic at time of diagnosis), choice of antifungal at diagnosis of IMI, antifungal exposure in the preceding 12 months before the diagnosis of IMI, site of infection, causative organism, minimum inhibitory concentration (MIC) reported (based on the Clinical and Laboratory Standard Institute [CLSI] broth dilution method) on sensitivity results, treatment course (antifungal[s] used and duration), clinical outcome, microbiologic outcome, need for surgical debridement, type of malignancy being treated, hematopoietic transplantation status (donor type), immunosuppressive medications in hematopoietic stem cell transplantation (HSCT) and engraftment status at the time of diagnosis with IMI, and the presence of graft versus host disease (GVHD). Patients without a diagnosis of culture-proven IMI caused by a resistant filamentous fungus, those who declined initiation of a terbinafine-containing combination antifungal treatment regimen, or those who documented allergy to terbinafine were excluded from the study.

This data was abstracted and analyzed over an approximately two-year period. Patients were deidentified and tracked by medical record number. Patient confidentiality was maintained by the exclusion of any specific protected health information such as names, birth dates, or other identifying information. As this was a retrospective study, a waiver of the Health Insurance Portability and Accountability Act (HIPAA) authorization from the IRB was obtained to permit the study team physicians to review patients’ medical charts to identify which patients would meet the study inclusion and exclusion criteria necessary to support the study’s research aims. There were no specific exclusion criteria save for ineligibility. The inclusion criteria were any patient treated for an IMI during the six-year study period. The sample size was based on patient availability and included all patients meeting the inclusion criteria. Data was analyzed by descriptive statistics. Comparisons were made between the two groups utilizing standard statistical significance criteria.

## Results

A total of 64 patients were included in the analysis using the above criteria. Findings are summarized in Table 1. The median age and gender in both groups were similar (p = 0.8). Causative organisms in both groups were primarily *Fusarium *species infections in this study. In the terbinafine treatment group (n = 14), there were four skin/soft tissue infections (29%), three rhinosinusitis (21%), another three pulmonary infections (21%), and four patients presented with either disseminated infection defined as multiple sites and/or an infection of unclear source (29%). In the control group not receiving terbinafine (n = 50), there were 14 skin/soft tissue infections (28%), eight patients had rhinosinusitis (16%), 19 had pulmonary infections (38%), and seven presented with an unclear source. Twenty-four patients in this group responded to the treatment and were later discharged (48%), 21 experienced treatment failure or died due to the IMI (42%), and five died from their underlying malignancy (10%).

**Table 1:** Demographics and patient characteristics (n = 64) Categorical variable comparison was done by descriptive statistics utilizing Fisher's exact test and Chi-square analyses. * Absolute neutrophil count < 500 cells/mm^3^. ^†^ Severe neutropenia for 2 weeks or more. ^‡^ *Syncephalastrum *spp., *Apophysomyces *spp., *Scopulariopsis *spp., *Phialemonium* spp., *Colletotrichum* spp., *Cunninghamella *spp., *Exophiala *spp., *Scytalidium *spp., and *Blastoschizomyces *spp. ^§^ Minimum inhibitory concentration.

Variables	Treatment Group A (n = 14)	Treatment Group B (n = 50)	p-value
Median age (IQR), years	64 (55, 69)	60 (48, 70)	0.8
Males, No. (%)	10 (71)	32 (64)	0.8
Severe neutropenia, No. (%)^*^	10 (71)	27 (54)	0.2
Prolonged severe neutropenia (%)^†^	11 (79)	28 (56)	0.3
Causative organisms, No. (%)			
*Fusarium *spp.	10 (71)	17 (34)	
*Aspergillus *spp.	1 (7)	17 (34)	
*Curvularia *spp.	0 (0)	3 (6)	
*Rhizopus* spp.	0 (0)	3 (6)	
*Mucor *spp.	0 (0)	1 (2)	
*Scedosporium *spp.	0 (0)	1 (2)	
Others^‡^	3 (21)	8 (16)	
MIC^§ ^to terbinafine ≥ 2 (n = 14)	3	11	
MIC to terbinafine < 2 (n = 19)	6	13	
MIC to terbinafine not reported (n = 31)	4	27	
Site of infection, No. (%)			0.5
Rhinosinusitis	3 (21)	8 (16)	
Skin and soft tissue infection	4 (29)	14 (28)	
Pulmonary	3 (21)	19 (38)	
Central nervous system	0 (0)	1 (2)	
Gastrointestinal	0 (0)	1 (2)	
Disseminated or unclear source	4 (29)	7 (14)	
Hematologic malignancy, No. (%)	12 (86)	42 (84)	
Solid organ malignancy, No. (%)	2 (14)	8 (16)	
Treatment outcomes, No. (%)			0.7
Discharged with a clinical response	5 (36)	24 (48)	
Treatment failure/died from IMI	8 (57)	21 (42)	
Died from underlying malignancy	1 (7)	5 (10)	

**Figure 1:**
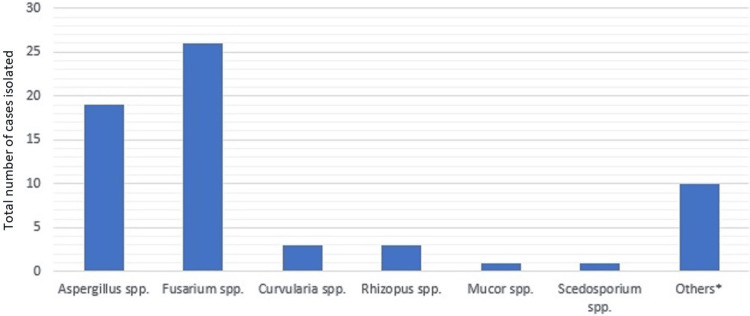
Distribution of causative agents in both treatment groups (n = 64) *Other isolates not otherwise specified in the major groups. These were often single or single-digit isolates. spp: Species.

The most common site of infection varied between the two groups, but the distribution of organisms in the two was not statistically dissimilar (p = 0.5). Outcomes were similar in terms of treatment response and secondary outcomes as well (p = 0.7). Prolonged neutropenia was significantly associated with worse outcomes (p = 0.0386). The distribution and frequency of pathogens are noted in Figure 1. There was no statistically significant improvement in outcome observed in this study when terbinafine was added to a combination regimen for the treatment of invasive mold infections.

It is worth noting that in these patients, terbinafine was added empirically before susceptibility results, given the lengthy delay for mold diagnosis and susceptibility testing. Unfortunately, substantial resistance in vitro to terbinafine was noted in several isolates. The small number of patients in the terbinafine group renders commentary on the outcomes when terbinafine yields an MIC < 2 compared to MIC > 2. However, it is strongly felt that timing is another factor with later initiation of a combination regimen unlikely to be successful if started late in the clinical course.

## Discussion

The question of utilizing multiple agents in the treatment of IMI is not new, and effective medical treatments remain a critical need for the immunosuppressed population. The standard of care, particularly in immunocompromised patients, is not clearly defined and oftentimes is institution-specific utilizing a standard operating protocol. Current guidelines continue to recommend single-agent therapy as the standard [7]. In complex cases such as those caused by mucormycosis, we continue to rely on surgical debridement or debulking of affected tissues, often a significantly morbid procedure for patients to undergo and potentially very dangerous in a cancer center’s patient population due to various laboratory abnormalities at baseline like thrombocytopenia. In this analysis, few infections were seen over the study period with fewer patients attempted on the terbinafine regimen. We did not appreciate a statistically significant difference in the outcomes studied.

Of note, there were fewer disseminated cases in the non-terbinafine group, implying the potential for the smaller number of patients in the study group to have been more severely ill, although this is not statistically significant. Aside from this, the most common site in both populations was the lungs. However, in the non-terbinafine group, there was a case of central nervous system (CNS) disease and a case of gastrointestinal (GI) disease, which was absent in the terbinafine group. While the low numbers make it difficult to generalize much from our study numerically, it is also noteworthy that a larger percentage of patients in the terbinafine group, almost two-thirds, had prolonged neutropenia (defined as severe neutropenia lasting two weeks or longer) compared to just over half of the patients in the non-terbinafine group. Some data suggest that the reversal of neutropenia itself in patients with IMI is the only factor independently associated with improved survival [13].

*Fusarium *species as mentioned in the results were the most common infectious agent in both groups. Additionally, only one case of aspergillus infection was noted in the terbinafine group along with a much greater distribution of overall infectious agents, which were largely not seen in the terbinafine group as well. It has been observed that a large proportion of our *Fusarium *species isolates that have been sent for sensitivity testing have returned with multiple antifungal drug resistance. In fact, the terbinafine group patients who had sensitivity data available reported that half of these cases resulted in a MIC to terbinafine ≥ 2. Unfortunately, almost half of all isolates from both groups had no sensitivity data available to compare, especially as it is not considered standard of care at the time of this manuscript submission.

Infections caused by drug-resistant fungi have become an increasingly concerning problem [14]. Resistance is the capability of organisms to grow in the presence of a medication that typically inhibits the growth of susceptible stains. Regulatory bodies such as the CLSI issue guidance on three standardized methods for measuring and interpreting the MIC of various medically relevant microbes (including fungi) to medications [15]. The term “clinical resistance,” which describes treatment failure despite the treatment with appropriate antifungal agents does not always correlate with in-vitro resistance. In this study, fungal resistance has to be taken to mean in vitro resistance. While standardized methods have been developed for broth dilution and disk diffusion testing of mold [15,16], there is still a need for further research to ascertain the correlation between in vitro data and clinical outcomes.

With the emergence of triazole-resistant strains of *Aspergillus*, there is data to show that infections with azole-resistant *Aspergillus* have been associated with a high risk of death and treatment failure [17,18]. There are an increasing number of case reports that similarly describe disseminated infections caused by *Fusarium* species [19]. Invasive infections with these resistant *Fusarium *isolates were linked with an estimated mortality rate of 50%-75% [20]. Due to the varying susceptibility profiles for *Fusarium *species, the optimal treatment option for disseminated infections caused by resistant strains is limited and controversial. In addition, there are no widely accepted guidelines for the treatment of infections caused by azole-resistant *Aspergillus *spp.

In-vitro experimentation suggests that the combination of terbinafine with certain azoles or the combination of terbinafine with liposomal AmB could have an additive to synergistic interaction leading to enhanced killing of *Aspergillus *species [11]. In-vitro studies have also suggested that combination therapy with certain azoles with terbinafine may have a synergistic effect against certain strains of *Fusarium *[21,22]. Furthermore, there is in-vitro data to show that combination therapy with terbinafine and itraconazole has synergistic activity versus *S. apiospermum* (other members of this genus of organisms have been recognized as having intrinsic resistance to several antifungal agents) [12,23].

While IMI remains a clear and present threat to the immunocompromised patients in our center, the incidence was lower than anticipated over our study period. Given the lower numbers, it is very difficult to draw conclusions through a direct comparison. However, our study represents the largest collection of patients in this population analyzed in this manner to date based on our literature searches. Further study of this agent across multiple centers may help clarify the role of this agent in IMI. Our internal conclusions by observation have also suggested that while the numbers statistically are low, the absolute numbers seem to suggest that the addition of terbinafine has not led to more favorable outcomes than in regimens sparing it. However, the addition of terbinafine appears to be tolerated without significant harm to the patients in any of our cases. Liver function tests (LFTs) were closely monitored during our chart review for consideration of safety, and we found no incidence of adverse events despite the addition of another agent. We submit that this agent is a potentially useful drug in our limited armamentarium against IMI.

## Conclusions

It remains clear that more effective agents are needed to reduce the morbidity and mortality of these devastating and potentially life-threatening infections. While newer agents are coming down the pipeline into clinical practice, such as fosmanogepix and orolofilm, it remains to be seen how effective these agents will be as monotherapy, and studies on combination therapy with these agents when they become available would also be useful. However, given the small number of cases available in a center such as ours, a multicenter study with a larger sample size may be necessary to further analyze the degree of efficacy of terbinafine more effectively than observational studies.
